# Cumulative Weight Exposure Is Associated with Different Weight Loss Strategies and Weight Loss Success in Adults Age 50 or Above

**DOI:** 10.1155/2015/904798

**Published:** 2015-06-23

**Authors:** Martin Sénéchal, Jana Slaght, Danielle R. Bouchard

**Affiliations:** ^1^Children's Hospital Research Institute of Manitoba, Winnipeg, MB, Canada R3E 3P4; ^2^Department of Pediatrics and Child Health, Faculty of Medicine, University of Manitoba, Winnipeg, MB, Canada R3E 3P4; ^3^Faculty of Kinesiology and Recreation Management, University of Manitoba, Winnipeg, MB, Canada R3T 2N2; ^4^Health, Leisure, and Human Performance Research Institute, Winnipeg, MB, Canada R3T 2N2

## Abstract

*Objectives*. To evaluate if cumulative weight exposure is associated with weight loss strategy choices and weight loss success. *Methods*. Data from the National Health and Nutrition Examination Survey were used; a total of 4,562 people age 50 years or older who reported trying to lose weight in the last year were studied. Cumulative weight exposure (CWE) score was defined as the sum of body mass index points above 25 kg/m^2^ at the age of 25, 10 years ago, 1 year ago, and now. Weight loss strategies were self-reported and weight loss success was defined as reaching a 5% weight loss in the last year. *Results*. Chosen strategies for weight loss vary across tertiles of CWE. Participants in the highest CWE tertile were about 4 to 20 times more likely to lose at least 5% of body weight in the past year compared to those in the lowest CWE tertile (*P* < 0.05).  *Discussion*. Strategies used to lose weight and weight loss success using different weight loss strategies vary considerably across cumulative weight exposure. Thus, cumulative weight exposure might be a variable worth considering when intervening with this population.

## 1. Introduction

The prevalence of obesity has increased in all age groups in the past decades [1–3]. There is a body of evidence showing that body weight usually increases in a linear fashion with age and peaks around 65 years [4]. As a result, the number of years spent carrying extra body weight will inevitably increase in the next generations as well as the prevalence of obesity in middle age adults and youth. Cumulative weight exposure (CWE) score which is defined as the sum of units of body mass index (BMI) over (positive) or under (negative) the upper limit of the optimal weight BMI category (25 kg/m^2^) for each of the available time points is one way to quantify the accumulation of weight over years [5, 6]. Prospective data from our group and others showed that CWE is associated with an increased likelihood of developing type 2 diabetes [5, 6], premature mortality [7, 8], and mobility impairment [9]. In addition, a recent study has reported that CWE was strongly associated with the increased likelihood of developing atherosclerosis in older adults [10]. These results suggest that CWE significantly impacts health in older adults and consequently it is imperative to study the implications of CWE for clinical settings.

The benefits of weight loss have been debated in older adults [11, 12]. However, a large randomized controlled trial of 585 older adults clearly demonstrated that the advantages of voluntary weight loss, using diet as a strategy, surpass the potential harms after 12 years of follow-up [13]. Regardless of that debate, more than 55% of adults over the age of 50 are trying to lose weight [14]. Although many strategies of weight loss are used, the first-line approach remains lifestyle modification including diet and exercise [15–17]. However, only 20% of obese individuals are currently able to maintain an initial 10% body weight loss for a period of two to five years using diet and exercise as the primary intervention [18]. Additionally, a recent study revealed that some weight loss strategies were more effective than others to reach a weight loss greater than 5% or 10% among obese adults [19].

It is likely that the CWE will increase in future generations of older adults and, so far, no one has verified if CWE is associated with weight loss strategy choices or if it is related to weight loss success. As a result, the first aim of this study was to evaluate whether weight loss strategy choices varied across different tertiles of CWE in older adults. The second objective was to investigate if CWE was associated with weight loss success (≥5% of total body weight between the self-reported weight 1 year ago and current body weight) stratified by weight loss strategies.

## 2. Methods

### 2.1. Study Population

The study sample consisted of 4,562 men and women aged 50 years and above who self-reported voluntarily trying to lose weight in the previous year in the U.S. National Health and Nutrition Examination Survey (NHANES: cycle 1999–2010). This cross-sectional study was completed in the noninstitutionalized population. Detailed survey operation manuals and consent forms are available on the NHANES website [20]. Briefly, the NHANES survey consisted of a home interview and a thorough health examination performed in a mobile exam center. During the home interview, participants were asked questions about their health status, disease history, and lifestyle behaviours. All participants provided written and informed consent. The protocol was approved by the National Center for Health Statistics.

Initially, 15,812 individuals were eligible based on age, but 3,877 were excluded because they were missing one or more time points for either body weight or height, precluding us from measuring CWE. In addition, those having a BMI below 18.5 kg/m^2^ (*N* = 273) at the tested cycle or the previous year were excluded from the dataset. Out of the remaining 11,616 individuals, only the 4562 individuals (39.3%) who reported trying to lose weight in the previous year were kept for the final analysis. Using Chi-square tests, no significant differences (*P* > 0.05) were observed for age (66.3 ± 10.1 versus 63.9 ± 9.2), sex (52% versus 54% women), ethnicity (non-Hispanic white 57.5% versus 58.5%; non-Hispanic black 18.8% versus 18.0%; Hispanic 20.8% versus 20.6%), income ≥ $55,000 (22.5% versus 23.0%), or education level (36.1% versus 34.6%) between participants included in the current study compared to the participants not included in this study.

### 2.2. Measurement of Cumulative Weight Exposure

Body mass was measured at the tested cycle but was self-reported for 1 and 10 years ago, as well as at 25 years of age. Height was self-reported at age 25 and measured at the tested cycle. BMI was calculated for each of the four time points. When calculating BMI at age 25, the self-reported height at age 25 was used. However, for calculating BMI 10 years ago, the mean between the self-reported height at age 25 and the measured height was used.

The CWE score was calculated as the sum of units of BMI over (positive) or under (negative) the upper limit of the optimal weight BMI category (25 kg/m^2^) for each of the available time points. For example, someone with a BMI of 26 kg/m^2^ at age 25, 23 kg/m^2^ 10 years ago, 32 kg/m^2^ last year, and 31 kg/m^2^ at the measured cycle was given a CWE of 12 (1-2 + 7 + 6). CWE was recently used by our group in a previous prospective study by considering the number of years between objective measures of body weight and height [6]. However, in the case of this study, it seemed imprudent to weigh each measure differently because the data on body weight and height were self-reported up to 60 years ago, and the exact years or the exact dates of measurement were not recorded.

### 2.3. Strategies for Weight Loss

All individuals who reported trying to lose weight the year prior to the measured cycle (in a previous question) were asked to give details about the strategies used. The question was how did you try to lose weight? This question was asked using an interviewer-administered computer-assisted personal interviewing system and completed at home. The participants have read all the options. In each option, the participants answered “yes” or “no” whether he or she tried to lose weight in the past year. A list of 14 to 20 options was given depending on the cycle. For cycles 1999 to 2004, 14 options were listed: eating less food, consuming less calories, eating less fat, doing exercise, skipping meals, eating diet food, eating a liquid diet, joining a weight loss program, using prescribed medication, using nonprescribed medication, vomiting, drinking more water, using a special diet (e.g., Atkins), or “others.” The participants could list as many “other” strategies as they wished. For cycles 2005 to 2010, six more options were available: eating fewer carbohydrates, smoking, consuming more fruits and vegetables, changing bad habits, eating less sugar, or seeking help from professionals. For analysis, individuals were given a value of 1 when answering positively to a strategy and a value of 0 when answering negatively to a strategy. Strategies reported by less than 5% of the sample were combined and labelled as “others.” The strategies include using prescribed medication, using nonprescribed medication, vomiting, smoking, and using a special diet. As a result, 14 individual strategies are described in Results in addition to the “other” variables.

### 2.4. Weight Loss Success

Weight loss success was measured as losing a minimum of 5% of total body weight between the self-reported weight 1 year ago and the current weight as proposed elsewhere [21]. In this study the amount of weight loss was calculated as the difference between self-reported weight one year ago and measured weight at the evaluation.

### 2.5. Confounding Variables

Covariates included in the analyses were age (continuous variable), sex, race/ethnicity (non-Hispanic white, non-Hispanic black, Hispanic, and other), smoking status (currently smoking or not), education level (<high school, high school, and college or more), family income (<$20 000, $20 001–$54 999, and >$55 000), and marital status (single or partner).

### 2.6. Statistical Analysis

Data management and statistical analyses were performed using SAS version 9.3 (SAS Institute, Carry, NC) and SPSS version 18. Because of the complex survey design used in NHANES, traditional methods of statistical analysis based on the assumption of a simple random sample are not applicable. Briefly, NHANES provides additional variables to be included in regression models to adjust for sample weights and complex survey design (strata, probability sampling units). In each regression model performed in the current study, three extra variables were added to the model. For weight, as indicated on the NHANES website, we used the smallest subpopulation that includes all the variables in the analysis (i.e., 2-year MEC exam weight [22]). For variance, the variables named variance pseudo-PSU and pseudostratum were added as indicated to the NHANES guidelines for variance [23]. More details are provided on their website [24]. Chi-square, one-way ANOVA, and general linear models were used to compare characteristics of our sample as well as the chosen weight loss strategies across tertiles of CWE. Linear regression models were used to test the association between the continuous CWE and each of the reported weight loss strategies. These models were adjusted for age, sex, race/ethnicity, smoking status, family income, marital status, education level, dataset weight, dataset variance, and BMI. Thereafter, logistic regression models were performed to investigate the likelihood of using different strategies of weight loss across tertiles of CWE. In addition, we performed logistic regression models to investigate if the CWE was an independent predictor of weight loss success. All the logistic regression models were adjusted for age, sex, race/ethnicity, smoking status, family income, marital status, education level, dataset weight, dataset variance, and either obesity status or BMI. Significance was set at *P* < 0.05.

## 3. Results

Table 1 describes general characteristics of the sample stratified by tertiles of CWE. There was a significant difference among tertiles of CWE for age, sex, obesity status, race/ethnicity, annual income, and marital status (all *P* < 0.05), while no difference was observed among groups education level and current smokers.

Table 2 shows that eating less food (68.7%) and doing more exercise (46.2%) were the most reported weight loss strategies in older adults. Significant differences were observed across tertiles of CWE for 8 out of the 15 possible self-reported strategies. However, only four strategies across CWE tertiles were significant after adjustments for confounders: exercising more (*P* = 0.01), drinking more water (*P* = 0.04), consuming more fruits and vegetables (*P* = 0.04), and eating fewer carbohydrates (*P* = 0.02).

Linear regression models were used to test the association between the continuous CWE and each of the reported weight loss strategies. The results showed that the same four strategies (i.e., exercising more, drinking more water, consuming more fruits and vegetables, and eating fewer carbohydrates) were associated with the CWE expressed as a continuous variable (*P* < 0.05) once adjusted for all confounders, including BMI.

**Figure 1:**
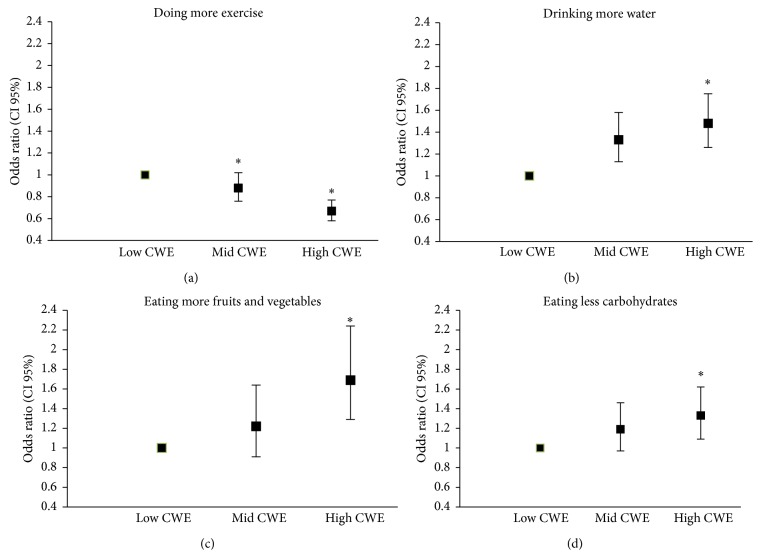
Strategies used to lose weight across CWE groups. ^*∗*^Significantly different from the low CWE group.

Figures 1(a), 1(b), 1(c), and 1(d) describe the likelihood of exercising more, drinking more water, eating fewer carbohydrates, or eating more fruits and vegetables as strategies to lose weight across CWE tertiles. Older adults in the highest tertile of CWE were 42% less likely to use exercise as a strategy to lose weight OR (95% CI) 0.58 (0.67–0.77). On the other hand, older individuals in the highest tertile of CWE were 1.48 (1.26–1.75) times more likely to drink more water, 1.69 (1.29–2.24) times more likely to eat more fruits and vegetables, and 1.33 (1.09–1.62) times more likely to reduce carbohydrates consumption, as strategies to lose weight compared with the low tertile of CWE (*P* = 0.01).

Table 3 represents the odds of achieving at least 5% body weight loss during the past year (≥5% of total body weight between the self-reported weight 1 year ago and the current body weight). When adjusted for obesity status, age, sex, race/ethnicity, smoking, household annual income, and marital status older adults in the highest CWE were 13.74 (95% CI: 7.23–26.14; *P* < 0.01) times more likely to do so if they increased exercise level as strategy compared with their counterparts in the lowest CWE group. The odds increased to 19.66 (95% CI: 9.60–40.23; *P* < 0.01) when using BMI instead of the obesity status in the model. Older adults in the highest CWE are 5.00 (2.29–8.99) times more likely to reach at least 5% weight loss if they increased water consumption and 8.82 (3.20–24.26) times more likely if they decreased carbohydrates, but not more likely to increase consumption of fruits and vegetables. The same significance level was observed when using BMI instead of obesity status in models. The respective numbers were 3.84 (1.71–8.64), 4.61 (1.70–12.52), and 2.22 (0.48–10.29).

## 4. Discussion

This study revealed novel insights into the management of obesity among adults age 50 or above. The first novel finding of the current study is that CWE is associated with the choice of weight loss strategies among adults age 50 years old and above. More specifically, people with the highest CWE are more likely to choose diet strategies such as drinking more water, eating fewer carbohydrates, and eating more fruits and vegetables and less likely to choose exercise as a strategy to achieve weight loss. The second novel finding is that CWE is associated with weight loss success in the past year when using these three specific strategies to lose weight. Collectively, these findings suggest that adults 50 years old or above who have accumulated extra body weight over the years might choose different weight loss strategies, and their weight loss success might differ based on the level of CWE.

As previously mentioned, reducing caloric intake and increasing exercise levels are the cornerstones for successful weight loss [15–17]. Based on the literature, the most popular weight loss strategy is to take fewer calories and eating smaller portions (less food) [14, 16, 19]. In the current study, when considering confounders, eating less food was not a weight loss strategy associated with CWE. This suggests that eating less food might be a strategy that adults are doing in general; however, our results support that older adults having accumulated extra body weight over the years are not more or less likely to decrease food portions in order to achieve weight loss. On the other hand, our results show that CWE was associated with two specific diet strategies to lose weight (i.e., drinking more water and reducing carbohydrates) even once adjusted for current obesity status or current BMI. This could be explained by the fact that older adults who have accumulated more excessive weight have already tried the most popular diet strategies (e.g., reducing fat, reducing calories, and increasing fruits and vegetables consumption) and are now looking for a new way to induce weight loss. To support this hypothesis, a study done with 20,000 people reported that BMI was positively associated with the number of attempts to lose weight in older adults [25]. In our study BMI was associated with the CWE (*r* = 0.78, *P* < 0.01) increasing the challenge to distinguish if a greater CWE was associated with weight loss strategies to reach 5% of weight loss in the past year independent of BMI. We tried to address this issue by using obesity status (Yes/No BMI ≥ 30 kg/m^2^). When logistic regression models to predict if doing more exercise, drinking more water, and eating fewer carbohydrates were associated with weight loss equal or above 5% in the past year, the high CWE group was more likely to have used these weight loss strategies compared with the low CWE group regardless of being adjusted for obesity status or BMI.

Previous studies have reported that between 50 and 60% of people are using exercise as a weight loss strategy [15, 16, 19], while in adults age 50 or above, the prevalence is 56.3% [15]. In our study, we observed a lower proportion (46%) of older adults in the same range of age using exercise as a strategy to lose weight. This difference could be explained in part by the fact that studies from the literature analysed data collected between 1998 and 2006, while our study took the data from 1999 to 2010. Consequently, it is possible that using exercise as a strategy to lose weight has declined as a result of the reduction of exercise levels in the whole population in the past few years [26], or as a result of the popularity of new weight loss strategies [27]. Even though our results confirm that exercise is a popular strategy chosen to lose weight among adults in that age range, it also extends the current knowledge by showing that if someone has accumulated weight throughout their lifespan, he or she is less likely to choose this strategy to lose weight. CWE has been associated with chronic conditions [7, 8] and mobility impairments [9] in the past. This could help explain why we observed that people in the highest CWE category reported that they were less inclined to choose to exercise in order to achieve weight loss.

In terms of weight loss success, our results show that adults age 50 years old or above who choose at least one of these strategies: exercise more, eat less carbohydrates, and drink more water, have four to twenty times greater odds of losing a minimum of 5% body weight in the past year if they have accumulated high excessive weight over the years. These findings are in line with strategies associated with losing a minimum 5% body weight reported by Nicklas et al. [19] who reported using a sample of adults that was associated with exercising more and using prescription weight loss medications. This latter difference might be explained by the fact that prescribed weight loss medications for older adults is still not widespread because of the possible risk of medication interactions, poor adherence to drug prescription [28], and the fact that weight loss drugs are usually not covered by health insurance which can cause a financial burden. In addition, our study includes data since 1999, when medications prescribed for weight loss were scarce, especially for older adults.

It has been demonstrated in the past that diet compliance is more important than the strategy to reach caloric reduction and weight loss [29]. However, our study suggests that some weight loss strategies might be more effective in a subsample of the population. For example, our study suggests that choosing to increase water consumption as a strategy to lose weight is three times more effective for individuals that have accumulated more weight over the years, compared with those who have accumulated less weight over the years, in achieving a minimum of 5% weight loss. Similar findings were observed for increasing exercise level and decreasing carbohydrates. Consequently, CWE might help to determine which adults would be more likely to respond positively to a given weight loss attempt using specific strategies.

Our current study revealed interesting results; however, some limitations need to be mentioned. The exposure variable is mostly based on self-reported measures of body weight and height. It is possible that body weight was underestimated and height overestimated as commonly reported [30, 31], especially that some individuals self-reported their body weight up to 60 years ago. One limitation of this study is that the CWE score was not weighed based on the number of years that one would have accumulated BMI below or above 25 kg/m^2^. We decided not to do this because of the weak association between objective and self-reported weight and height measurements, especially when the self-reported measures go up over the decades [31–34]. Because of the strong association between the CWE and BMI, it is impossible to test both variables in the same model and determine what variable is more associated with the studied outcomes. The interaction between different self-reported weight loss strategies was not evaluated in the current study. It is possible that participants used combinations of different strategies to attempt weight loss, and perhaps changing one strategy such as increasing water consumption solely might not be associated with weight loss. Three cycles provided 19 options of weight loss strategies plus a section called “others,” while three cycles (1999–2004) provided only 13 strategies plus a section called “others.” Even if the participants could have reported any other strategies in “others” from 1999 to 2004, less than 1% of the sample reported anything in that category. This is not surprising considering that some literature reports that the use of open questions versus choosing from a list of answers leads to different answers [35]. Thus, it is possible that the strategies reported in efforts to lose weight in the past year could have been different if the possible answers had been the same in all the cycles. The cross-sectional design of this study limits our observation to association conclusions instead of causal effect. This study included evaluation done between 1999 and 2010, so it is possible that reported weight loss strategies were influenced by different temporal trends. Also, even if a person reported using a specific strategy to lose weight in the past year, we have no method to measure if the strategy was used and to what extent. Finally, we were not able to quantify the exact time between measures.

## 5. Conclusion

CWE score is associated with the use of different weight loss strategies among adults age 50 years old or above. In addition, CWE is a determinant of weight loss success when using specific weight loss strategies among that population. Clinical trials are now needed to identify if CWE is associated with weight loss success when weight loss strategies are controlled and weight loss is objectively measured.

## Figures and Tables

**Table 1:** Characteristics of the 4562 study participants.

	Low CWE ≤6.56	Mid CWE 6.57–20.86	High CWE ≥20.87	*P* value
General characteristics				
Age (years)	64.2 ± 9.6	64.6 ± 9.2	62.9 ± 8.6	<0.01
Men (%)	550 (36.2)	820 (53.9)	730 (48.0)	<0.01
BMI (kg/m^2^)	26.3 ± 2.5	30.8 ± 2.6	37.8 ± 5.7	<0.01
Obesity status				
Obese at age 25	3 (0.2)	19 (1.2)	279 (18.3)	<0.01
Obese 10 years ago	11 (0.7)	339 (22.3)	1239 (87.5)	<0.01
Obese 1 year ago	96 (6.3)	1009 (66.3)	1496 (98.4)	<0.01
Currently obese	94 (6.2)	907 (59.6)	1466 (96.4)	<0.01
Successful weight loss	502 (33.0)	684 (45.0)	842 (55.4)	<0.01
Race/ethnicity				0.01
Non-Hispanic white	1006 (66.2)	852 (56.0)	767 (50.4)	
Non-Hispanic black	174 (11.4)	281 (18.5)	404 (26.6)	
Hispanic	272 (17.9)	357 (23.5)	319 (20.9)	
Other	68 (4.5)	31 (2.0)	31 (2.0)	
Education level				0.61
Less than high school	636 (41.8)	624 (41.0)	616 (40.5)	
High school	339 (22.3)	318 (20.9)	349 (20.9)	
College or above	533 (35.1)	565 (37.1)	547 (36.1)	
Smoking level				
Currently smoking	323 (21.3)	327 (21.1)	316 (20.8)	0.99
Household annual income				0.01
≤$20,000	314 (20.7)	336 (22.1)	386 (25.4)	
$20,000–54,999	573 (37.7)	556 (36.6)	597 (39.3)	
≥55,000	374 (24.6)	354 (23.3)	397 (19.5)	
Marital status				0.02
Married/partner	805 (53.0)	858 (56.4)	784 (51.5)	

Data are presented as mean (SD) for continuous variables and *n* (%) for categorical variables.

CWE = cumulative weight exposure.

**Table 2:** Reported strategies to lose weight in the past year.

Strategies	Prevalence	Low CWE	Mid CWE	High CWE	*P* value
Less food	3132 (68.7)	1041 (68.5)	1013 (66.6)	1078 (70.9)	0.04
Less calories	1651 (36.2)	551 (36.2)	531 (34.5)	569 (37.4)	0.36
Less fat	1978 (43.4)	334 (9.0)	616 (15.5)	1028 (25.9)	0.19
Less sugar	264 (5.8)	80 (5.3)	86 (5.7)	98 (6.4)	0.36
Less carbohydrates	732 (16.0)	217 (14.3)	242 (15.9)	273 (17.9)	0.02^*∗∗*^
Skip meals	663 (14.5)	177 (11.6)	213 (14.7)	263 (17.3)	0.19
Diet food products	404 (8.9)	116 (7.6)	133 (8.7)	155 (10.2)	0.19
Drink more water	1247 (27.3)	361 (23.8)	414 (27.2)	472 (31.0)	0.04^*∗∗*^
Liquid diet	229 (5.0)	87 (5.7)	74 (4.9)	68 (4.5)	0.27
More fruits and vegetables	336 (7.4)	87 (5.7)	105 (6.9)	144 (9.5)	<0.01^*∗∗*^
Modify bad habits	219 (4.8)	59 (3.9)	70 (4.6)	90 (5.9)	0.03
Join a weight loss program	260 (5.7)	80 (5.3)	75 (2.9)	105 (6.9)	0.04
More exercise	2106 (46.2)	756 (49.7)	715 (47.0)	635 (41.7)	<0.01^*∗∗*^
Consult health professional	265 (5.8)	60 (3.9)	89 (5.9)	116 (7.6)	0.04
Others^*∗*^	287 (6.3)	87 (5.7)	93 (6.1)	107 (7.0)	0.31

Data are presented as *n* (%); Chi-square tests were used across tertiles of CWE.

*P* value is unadjusted.

^*∗*^Others included using prescribed medication, using of nonprescribed medication, vomiting, smoking, and using a special diet.

^*∗∗*^Remained significant once adjusted for confounders.

CWE = cumulative weight exposure.

**Table 3:** Odds of losing at least 5% of body weight in the past year.

	Low CWE	Mid CWE	High CWE
Doing more exercise			
** **Model with obesity status	1.00	3.27 (2.10–5.09)	13.74 (7.23–26.14)
** **Model with BMI	1.00	3.69 (2.30–5.94)	19.66 (9.60–40.23)
Drinking more water			
** **Model with obesity status	1.00	1.92 (1.06–3.48)	5.00 (2.29–10.94)
** **Model with BMI	1.00	1.41 (0.80–2.49)	3.84 (1.71–8.64)
Eating less carbohydrates			
** **Model with obesity status	1.00	3.62 (1.76–7.44)	8.82 (3.20–24.26)
** **Model with BMI	1.00	2.53 (1.26–5.09)	4.61 (1.70–12.52)
Eating more fruits and vegetables			
** **Model with obesity status	1.00	2.10 (0.57–7.69)	3.02 (0.62–14.68)
** **Model with BMI	1.00	1.48 (0.45–4.92)	2.22 (0.48–10.29)

Odds ratio (95% confidence interval).

All models were adjusted for age, sex, race/ethnicity, smoking status, family income, marital status, education level, dataset weight, dataset variance, and either obesity status or BMI.

CWE = cumulative weight exposure.
